# Increasing the scale and adoption of population health interventions: experiences and perspectives of policy makers, practitioners, and researchers

**DOI:** 10.1186/1478-4505-12-18

**Published:** 2014-04-15

**Authors:** Andrew J Milat, Lesley King, Robyn Newson, Luke Wolfenden, Chris Rissel, Adrian Bauman, Sally Redman

**Affiliations:** 1 Centre for Epidemiology and Evidence, NSW Ministry of Health, Locked Mail Bag 961, North Sydney, NSW 2059, Australia; 2 School of Public Health, University of Sydney, Level 2, Medical Foundation, Building, K25, Sydney, NSW 2006, Australia; 3 School of Medicine and Public Health, University of Newcastle, University Drive, Callaghan, NSW 2308, Australia; 4 Sax Institute, Level 2, 10 Quay, St, Haymarket, NSW 2000, Australia

**Keywords:** Intervention development, Intervention studies, Scaling up

## Abstract

**Background:**

Decisions to scale up population health interventions from small projects to wider state or national implementation is fundamental to maximising population-wide health improvements. The objectives of this study were to examine: i) how decisions to scale up interventions are currently made in practice; ii) the role that evidence plays in informing decisions to scale up interventions; and iii) the role policy makers, practitioners, and researchers play in this process.

**Methods:**

Interviews with an expert panel of senior Australian and international public health policy-makers (n = 7), practitioners (n = 7), and researchers (n = 7) were conducted in May 2013 with a participation rate of 84%.

**Results:**

Scaling up decisions were generally made through iterative processes and led by policy makers and/or practitioners, but ultimately approved by political leaders and/or senior executives of funding agencies. Research evidence formed a component of the overall set of information used in decision-making, but its contribution was limited by the paucity of relevant intervention effectiveness research, and data on costs and cost effectiveness. Policy makers, practitioners/service managers, and researchers had different, but complementary roles to play in the process of scaling up interventions.

**Conclusions:**

This analysis articulates the processes of how decisions to scale up interventions are made, the roles of evidence, and contribution of different professional groups. More intervention research that includes data on the effectiveness, reach, and costs of operating at scale and key service delivery issues (including acceptability and fit of interventions and delivery models) should be sought as this has the potential to substantially advance the relevance and ultimately usability of research evidence for scaling up population health action.

## Introduction

To maximise the impact of public health research, interventions found to be effective in improving health need to be scaled up and delivered on a population-wide basis. The transfer of new knowledge from public health research into practice, however, continues to be sub-optimal [1]. On average, it takes over 6 years for research evidence to reach reviews, papers, and textbooks, and a further 9 years for this evidence to be implemented into practice [2]. Both the failure of effective public health initiatives to influence public health practice and the lag between evidence generation and implementation represent considerable impediments to population health improvement as it denies or delays community access to effective services [3-5].

Scaling up is the process by which health interventions shown to be efficacious on a small scale and/or under controlled conditions are expanded under real world conditions into broader policy or practice [6,7]. Understanding how policy makers and practitioners make decisions about whether to scale up interventions and the role of evidence in these decisions, may facilitate more complete and timely translation of research into practice. This issue of how best to scale up health interventions has been receiving some recent attention, particularly in the global health literature [6,8-13] and through case studies [8,10,12,14,15]. However, there are few studies in high-income countries.

The concept of scaling up is different from routine adoption as it involves an explicit intent to expand the reach of an intervention to new settings or target groups. Norton and Mittman [11] examined key barriers and facilitators to scaling up of 10 disease prevention programs in the United States using interviews with program representatives. Key barriers to scaling up included reluctance by implementing organisations to fully integrate programs into routine service delivery on top of existing workloads and a lack of resources to implement programs with fidelity or at all in “real-world” settings, particularly when the efficacy trials generating the evidence had been expensive. Key success factors included sustained involvement of highly committed individuals and the development of scaled up programs in community settings, rather than more traditional researcher-initiated and managed programs.

Similarly, a literature review and two-stage Delphi process with 14 senior Australian population health policy makers and researchers [6,9] concluded that scaling up can be aided by producing information on effectiveness, reach, and adoption, human, technical, and organisational resources, costs, intervention delivery arrangements, contextual factors, and applying appropriate evaluation approaches. This study concluded that if these “scalability considerations” are addressed in the funding, design, and reporting of intervention research, it could advance the quality and usability of research for decision makers, and by so doing, improve uptake and expansion of promising programs into practice [6].

Though these recent studies, made up primarily of case studies, literature reviews and interviews with policy makers and practitioners [6,11,12,16] describe principles of scaling up of health interventions, none have specifically examined how scaling up decision processes actually occur in the real world using respondents actively involved in population health scaling up decision processes. Furthermore, there remains an absence of a comprehensive and systematic description of the respective roles that policy makers, practitioners, and researchers play in these processes. The empirical study of these factors is important on a number of levels. Firstly, the failure to account for differing decision-making processes and incentives for researchers, policymakers, and practitioners has been purported as a major contributor to the lack of consistent and systematic transfer of research evidence into broader policy and practice [16,17]. Such research also provides a more pragmatic way of understanding how research can be used, grounded in real policy making processes, rather than idealised accounts of how this could occur.

In light of these gaps in our knowledge, the objectives of the current study were to examine:

i) how decisions to scale up population health interventions in high-income country contexts are made in practice;

ii) the role that research evidence plays in informing decisions to scale up promising interventions; and

iii) the roles policy makers, practitioners, and researchers play in the process of scaling up population health action.

## Methods

### The selection of experts

Purposeful sampling techniques were used as expert opinions were sought from senior policy makers, practitioners, and researchers [18]. Experts invited to participate in the study comprised current and former senior government and non-government policy makers, selected by the study investigators for their extensive experience in commissioning and or implementing large scale population health programs at state, national, or international levels; senior practitioners/service managers selected for their extensive experience in the on-the-ground implementation of local population health programs; and senior researchers, mostly Associate Professor or above, selected for their extensive experience as chief investigators of applied and intervention research. Policy makers were employed by a mix of state-wide, national, and international policy making agencies. Where experts were employed as policy makers or practitioner/service managers and simultaneously held academic appointments, respondents were categorised as either policy makers or practitioners. The sample of experts were selected mainly from Australia, but also from Asia, United Kingdom, and the United States. Using similar health promotion studies seeking expert opinion as a benchmark, the current study aimed to recruit approximately 20 respondents [19].

### Interviews

Interviews were conducted by a research assistant with over 10 years’ experience in the conduct of qualitative methods and included a mix of open and closed questions to assess how these decision makers had made decisions to scale up interventions in practice, how evidence had informed their decisions and the respective roles policy makers, practitioners, and researchers had played in these scaling up processes. The interview questions addressed the aforementioned gaps in the knowledge base identified in previous research [6]. All interviews were recorded with the consent of participants using a digital recorder and later transcribed. For international respondents the interview rounds used the same questions, but were completed as self-administered surveys. The interview questions used in the study are shown below.

### Interview/survey questions

1. How many years’ experience do you have in the development, implementation, and or evaluation of population health interventions? [Years]

2. What is your current role? [Policy maker; Researcher; Both policy maker and researcher; Practitioner/Service Manager; Consultant; Other]

3. Have you ever been involved in decision processes to scale up population health interventions into broader policy or practice?

(*Prompt, if required: By scaling up we mean taking a health intervention shown to be efficacious on a small scale and or under controlled conditions and expanding it under real world conditions into broader policy or practice. Decision processes are processes by which decision makers identify information, evaluate alternatives, and make decisions on courses of action*.) [Yes/No]

4. In the past five years, how many times have you been involved in decision processes to scale up population health interventions? [Times]

Now, thinking specifically about the last time you were involved in a decision process to scale up a population health intervention.

4a. Could you briefly describe the intervention?

4b. What formal processes, if any, were undertaken to inform decisions to scale up the intervention (or not)? (*Prompts if required: stakeholder consultation, expert consultation, Advisory Group, meetings of Ministers, etc.*)

4c. What role did you personally have in the decision processes? (*Prompts if required: Policy maker, advocate, expert, advisor, etc.*)

4d. How or by whom was the final decision to scale up the intervention (or not) made? (*Prompts if required: political process, policy process, an individual such as a Minister or senior bureaucrat, by a Government/s, etc.*)

4e. What (if any) influence did research evidence have on the decision processes?

4f. If research evidence did influence the decision processes, what type of research was used and how was it applied? *(Prompt if necessary: descriptive epidemiology, determinants research, formative research, measurement research, intervention research, economic evaluation, etc.*)

4g. What factors and information sources, if any, enabled decision-making about whether to scale up the intervention or not?

4h. What factors were barriers to decision-making about whether to scale up the intervention or not?

4i. What were the final outcomes of these decision processes? (*Prompt if necessary: intervention was scaled up, intervention abandoned, etc.*)

4j. Was this process typical? If not, can you describe how it differed from other processes (if any) you have been involved in previously.

5. From your experience, what roles do policy makers and researchers play in process of scaling up population health interventions? (*Prompts in required: how do they differ and why?*)

6. From your experience, what are the most powerful influences on decisions to scale up population health interventions? In your opinion, which should be the most important influences on decisions to scale up population health interventions?

Now, thinking about operationalizing decisions to scale up population health interventions into broader policy and practice.

7. Have you ever been responsible for implementing a process of scaling up a population health intervention into broader policy or practice? (*Prompt, if required: by scaling up we mean taking a health intervention shown to be efficacious on a small scale and/or under controlled conditions and expanding it under real world conditions into broader policy or practice.*) [Yes/No]

8. From your experience, what do you think are the key barriers to effectively scaling up population health interventions into broader policy and practice?

9. From your experience, what do you think are the key success factors in scaling up population health interventions into broader policy and practice?

### Data analysis

The lead author and research assistant collated responses to quantitative survey items. A two-stage qualitative ‘thematic analysis’ method [20] was used to interpret responses to open-ended questions. Thematic analysis is a widely used method for identifying, analysing, and reporting patterns (themes) within data [21]. In this study, thematic analysis involved the lead author and research assistant independently assessing transcribed responses to identify broad coding themes for each question. These independently generated draft coding frames were tabled and discussed and final agreement on the code frame was reached. This process was followed by a final joint thematic analysis of the data.

### Ethical approval

The study received Low and Negligible Risk Ethical approval from Royal Prince Alfred Hospital Research Office Protocol No X11-0024 & HREC/11/RPAH/33.

## Results

### Response rates and respondent characteristics

Of the 25 invited experts, 21 agreed to participate (84% response rate), with 7 of 8 policy makers, 7 of 7 practitioners/service managers, and 7 of 10 researchers approached agreeing to participate. Policy-makers (n = 7) possessed a mix of senior policy experience at the state, national, and international levels, and had a mean of 18 years’ experience in commissioning and/or conducting major intervention research trials and overseeing implementation of large-scale population health programs. Practitioners/service managers possessed extensive service development experience at local and regional levels and had a mean of 17.4 years’ experience in overseeing intervention research trials and expanding the implementation of prevention programs. The sample of researchers (n = 7) was a mix of Australian and international University based experts in public health, health services, and health promotion research with a mean of 22.1 years of experience. Four of the 21 respondents held senior public health or academic positions in Asia, Europe, or the United States.

### Involvement in the decision-making processes and implementation of scaling up processes

All of the policy makers and practitioners and 6 of the 7 researchers interviewed had been or were currently involved in decisions about whether to scale up population health interventions into policy and practice.

Respondents were also asked how many times in the past 5 years they had been involved in decision processes to scale up population health interventions. Researchers most frequently reported involvement in between 1 and 6 cases and policy makers and practitioners most frequently reported involvement in 10 or more and 6 or more cases of decision processes, respectively.

All of the policy makers and practitioners interviewed had been responsible for implementing scaled-up interventions into policy and practice, while 2 of 7 researchers had experienced such a responsibility.

### The type of interventions scaled up

Respondents were asked to describe the most recent intervention for which they were involved in scaling up related decision processes (Table 1). The 20 interventions described by respondents included lifestyle behaviour change, organisational change, brief intervention counselling, social marketing, and sponsorship. They addressed issues such as healthy eating, physical activity promotion, chronic disease prevention and management, diabetes prevention, and alcohol and tobacco control. The scaled-up interventions were implemented in a variety of settings, focusing on either children or adults. Interventions were implemented mainly in Australia, but also in the pacific region, United States, and South America.

**Table 1:** The type of interventions that were scaled up into broader policy and practice

**Health issue**	**Intervention type**	**Setting**	**Target population**	**Size of intervention**	**Country**
1. Healthy eating	Policy & practice change in Canteens	Schools	Children	State-wide	Australia
2. Physical activity	Brief intervention	Primary care	Adults	Local	Australia
3. Physical activity	Exercise classes	Community	Adults	National	Brazil
4. Healthy eating & physical activity	Policy & practice change and provider training	Childcare	Children	Local	Australia
5. Healthy eating & physical activity	Lifestyle modification program	Community	Adults	State-wide	Australia
6. Healthy eating & physical activity	Policy & practice change and curriculum support	Schools	Children	State-wide	Australia
7. Healthy eating & physical activity	Policy & practice change and provider training	Childcare	Children	Local	Australia
8. Diabetes	Lifestyle modification program	Primary Health Care	Adults	Local	Australia
9. Healthy eating and physical activity	Whole of school policy & practice change	Schools	Children	National	Fiji
10. Healthy weight	Lifestyle modification program	Community	Children	State-wide	Australia
11. Chronic disease management	Lifestyle modification program	Community	Adults	State-wide	Australia
12. Chronic disease risk factors	Brief interventions and referral	Community health services	Adults	Local	Australia
13. Chronic disease risk factors	Brief interventions	General practice	Adults	National	Australia
14. Chronic disease risk factors	Brief intervention and referral	Community health services	Adults	Local	Australia
15. Chronic disease risk factors	Policy, practice change, workforce development	Local government/community	Adults and children	State-wide	Australia
16. Diabetes prevention	Lifestyle modification program	Health services	Adults	National	USA
17. Diabetes prevention	Lifestyle modification program	Primary health services	Adults	Local	Australia
18. Tobacco cessation	Mass media campaign	Community	Adults & young people	State-wide	Australia
19. Binge drinking	Government sponsorship of sport and social marketing campaign	Community	Young people	National	Australia
20. Falls prevention	Website & group based exercise/education program	Community	Adults	State-wide	Australia

### In-depth interviews

#### Scaling up decision processes

Respondents across professions reported that formal scaling up processes were generally iterative, and were run by policy makers and or practitioners: *‘It was an iterative process. We got technical expert advice from the market about the do-ability …which was very important because it was all very well coming up with the idea but without the practical, this is how much it will cost and yes it’s available, you can do it or you can’t do it, it was all kind of moot*… *It was also important for our minister to be able to say we had that consultation and we had that credibility with the public health community’* (PM).

Policy makers described the process of constructing a case for action for the consideration and endorsement of political leaders and senior executives in the form of parliamentary, ministerial, and executive briefings. Policy makers rarely made decisions in isolation and without first seeking senior endorsement of proposed approaches and associated budgets from senior delegates. One senior policy maker described the process as follows: *‘The Minister is actually making a decision to fund a program. It’s not a conscious decision that they are scaling up… from research evidence into a state-wide program… The decision is about what advice policy makers give to the Minister and based on this advice the Minister makes a decision about whether to fund the program.’* (PM); and *‘…ultimately we had to take it to our minister as a package proposal’* (PM).

It was observed that decisions to scale up interventions were almost always subject to processes of either internal and/or external consultation through organizations and/or stakeholder networks, using advisory committees, working parties, expert advice, and often involving researchers either directly or indirectly: *‘We formed a clinical advisory group with clinicians to formulate the model of care. Then we rolled it out. We then formed partnerships with each of the, what we call, clusters, groups of community health services. We formed partnerships with their executives where they gave us local advice’* (P/SM).

Researchers agreed that while they might formulate recommendations, ultimate decisions to scale up interventions were generally made by policymakers and practitioners within government agencies, over which they had little control. Researchers reported that they collated and provided evidence to others in a number of forms and forums, including systematic reviews, intervention research results and expert advice, but they were not always sure how that evidence was then used by others to inform the decisions that were made about scaling up the intervention: ‘*I was consulted around interpreting the findings and being asked to give my opinion about recommendations for the future, but how those were considered with the processes that were followed to make the final decision internally I don’t know’* (R).

Some researchers noted that their role in decision-making processes went beyond providing evidence, to one of advocating for particular approaches: *‘I was asked to sit on the steering committee for this play space initiative and then it was through that that I put forward or really advocated for consideration of our evidence-based intervention as part of the fleet of programs, chronic disease prevention and management programs that would be scaled up and delivered’* (R).

There was consistency in the view that the context for decisions about whether to scale up interventions is highly political, rapidly changing, and influenced by a variety of factors, inputs, and relationships, including individuals’ values, skills, and experience. Respondents across occupational groups noted that the most powerful influences over scaling up processes were political and resource related: as one policy maker put it: *‘Politicians. One stroke of the pen, done’* (PM); and as a practitioner/service manager put it: *‘…obviously you need to have sufficient resources to enable it to happen, or at least have commitment that the resources will be available or can be attainable’* (P/SM).

#### The role of evidence in scaling up processes

Most decision-making processes associated with scaling population health interventions involved consideration of a variety of information sources, not just research evidence. Many respondents across occupation groupings observed that while research evidence was important, other contextual information or political influences also appeared to have a strong influence on the final outcome of decision processes. For example, alignment with government priorities and political imperatives, funding availability, leadership support, and support from stakeholders were thought to be particularly important influences by policy makers. Where research evidence was available, decisions were based on a body of evidence rather than a single study: *‘The government doesn’t just make decisions on the basis of one research project… but about the overall body of evidence… And so I think in terms of research evidence in general, I think it had quite a strong influence but it’s not just the only factor, so it’s a kind of necessary but insufficient condition’* (R).

A key theme identified by a number of policy makers acknowledged that research evidence was the starting point, but that: *‘…I knew that I had to take into account the policy context, the relationships we had politically with Ministers and the relationship we had between different sectors’* (PM).

Conversely, some policy makers suggested that from time to time decisions had been made without solid evidence, particularly where potential gains to the health of population are great: ‘*Plenty of critics were happy to say “well it’s never been done before, how do you know it will work?” That’s the reality of any large scale population-based intervention. Someone has to do it first. Someone had to legislate to make us wear seatbelts first’* (PM).

The types of research evidence reported as having been used in decision processes varied greatly, and comprised epidemiological data, intervention research, systematic reviews, controlled trials, and local quasi-experimental pilot studies. Policy makers and practitioners noted that epidemiological evidence was used to determine the nature and scope of a problem and for surveillance purposes: *‘Epidemiology or prevalence data provided a map or primary rationale for working in the setting’* (P/SM).

Respondents frequently reported the use of evidence from systematic reviews where these were available to build a case for scaling up the intervention: *‘We also used the evidence available from systematic reviews to argue that if the intervention was delivered, a risk reduction outcome could be achieved’* (P/SM).

Policy makers and practitioners identified a paucity of intervention research in the literature to inform scaling up efforts, as one policy maker put it: *‘The evidence provided us with clues to what might be appropriate to include… so the sorts of things about how many call backs were required…who should we be targeting, so there was no single research study but it was elements of other sorts of bits and pieces of programs that we needed to make a judgment call on’* (PM).

When intervention research was available, this evidence alone rarely provided policy makers with all of the information they needed to scale up interventions: *‘So few research studies provide the really nuts and bolts information that a policy maker would require’* (PM).

Research evidence was almost always adapted to context and situation. ‘Off the shelf’ interventions were generally not seen as appropriate by policy makers and practitioners unless they had been developed to suit the local context. Local evidence had a higher value because it was considered more likely to successfully translate into practice. Local evidence of efficacy or evidence from local pilots or replication studies was thought to be particularly persuasive: ‘*We used the local evidence we had gathered to show that the intervention could be delivered at no cost to service delivery and was acceptable to the service providers’* (P/SM).

A number of policy makers and practitioners observed that where direct evidence of effectiveness was not available, parallel evidence from other settings or health issues or countries was sometimes used: ‘*Then we also looked at, as I mentioned, other health topics, like so if we looked for research where there had been some up scaling, whether or not it was specific to our health topic or not, but specific to the setting…’* (P/SM). Both policy makers and practitioners spoke of drawing on the evidence in relation to the principles of successful interventions or best practice population health approaches and theories, in cases where sufficient evidence was not available.

Grey literature (including technical reports) was thought by policy makers and practitioners to be a particularly useful source of evidence as it often provided more ‘how to’ information than peer reviewed papers, although it could be difficult to access. Where this information was not available, advice and information was often directly sought from people who had implemented similar programs: *‘…a lot of the research is grey when it comes from other departments and closely held so that always makes it difficult. There’s a lot of searching out the project managers, the project workers and talking to them personally…what their perspectives were, how they actually make things happen on the ground and then what are the process outcomes…’* (PM).

Respondents across occupational groupings in the sample, but particularly policy makers, stated that evidence of implementation costs and cost effectiveness had been rarely available to them from the research literature, and yet were needed to inform scaling up processes: ‘*I think one of the barriers was the lack of costing information. One of the other barriers was not having clear cut cost effectiveness data on $1 spent here is going to give you $5 in return. We had evidence that it was effective, but we didn’t have evidence that it was cost effective’* (PM).

Many respondents across occupational groupings in the study sample observed that the value and importance of considering information in relation to the organisational fit of an intervention, and the acceptability and feasibility of the intervention to individuals and delivery agents: *‘…need to assess current practice. Determine the factors that influenced decisions in the setting. What the provider attitudes are, the way things are organized organisational/infrastructure issues. What service models and systems can be used - framework for understanding? Support for specific arrangements and links to other programs, business arrangements, workforce funding. These feasibility issues were researched so decisions could be made about how to scale up the intervention’* (R).

Scaling up processes that do not consider this information were seen by many respondents across occupation groupings as sub-optimal and more likely to meet system resistance, and less likely to be sustained in the longer term.

#### Different roles of policy makers, practitioners, and researchers

Respondents across the sample perceived that policy makers, practitioners/service managers, and researchers had different but complementary roles to play in the process of scaling up population health interventions.

##### Role of researchers

Researchers played an important role in bringing evidence to the attention of opinion leaders and decision makers, by providing independent expert opinion and by advocating for particular interventions or issues. *‘I think they have been particularly critical in my experience because they can be that expert independent voice that is needed sometimes that you can roll out. Basically, a face behind the paper or someone that can actually put a voice to a paper in front of a power broker or someone that holds power’* (P/SM). It was clear that researcher-generated information contributed to the way policy makers and practitioners thought about an issue.

Some researchers stated that they participated in discussions with policy makers about ‘how to’ implement, while others typically confine their advice to ‘what’ to implement. It was suggested that there were limited rewards for researchers to participate in scaling up decision-making processes, as one respondent noted: *‘…because I come originally from a practice background I am willing to kind of engage in broader discussions but I think a number of researchers would think that this is not their job’* (R).

Policy makers also noted that there were certain researchers who were well connected with the policy context and were willing to assist in scaling up decision processes: *‘There’s the researchers that keep their hand in, they’re the go-to people, the people I can pick up the phone to. “I’ve got this issue with this, have you got any evidence around this, what’s your feeling, have you done some research in this area” and they understood the context that I was talking about because we had worked together because I knew they were working in the same field, we all kind of understood each other and I knew what they were doing and I knew their strengths and capacities. Those were the go-to people’* (PM).

It was noted by a number of policy makers and practitioners that there was little evaluation conducted by researchers on scaled up interventions: *‘Well, my experience with researchers is that they never do research which is about scaling up. Research that’s about scaling up is probably less attractive to them than research that either proves something is effective or not…’* (PM)*.*

However, it was suggested by other respondents that there was scope for researchers to be engaged in ongoing evaluation for scaled up interventions: *‘…providing the capacity for ongoing evaluation, which may be part of the plan that the policy makers come up with’* (R). This opportunity may be particularly salient where practitioners had dual roles as implementers and researchers. In this regard it was suggested that researchers could ensure that intervention evaluations were being implemented with some rigor.

##### Role of policy makers

Policy makers play important roles in determining priorities, securing resources, and solidifying leadership and stakeholder support for action. They make decisions about what to implement and how to do it based on a variety of factors including the available evidence. Policy makers may also be involved in directing the implementation of interventions. As a researcher observed: *‘There are the political factors that they* [policy makers] *have to be aware of, in terms of what the political consequences may be of a program being rolled out more broadly or continued, to elected the person at the top of the tree. There are probably internal political factors that have also got to pay attention to, in terms of their organisation and support for an area of work, what the consequences might be for continuing to scale or deliver a program. They are very dependent on partnerships and therefore they have got to think about what the consequences are for the important partnerships of scaling up’* (R).

##### Role of practitioners/service managers

Practitioners/service managers in this study described a similar role to policy makers, but on a local level, as they sought executive endorsement of approaches to scaling up and had a particularly important role in building the relationships, systems, processes, and infrastructure that support the scaling up of population health interventions into practice. As one service manager put it: *‘So I guess I lead the process, as the service director so I initiated some of the earlier discussions, seeking feedback from key stakeholders and gaining that leadership support and developing those relationships to actually support the process and then basically I developed the briefs, etc. to get it into the level. I had those high level decision meetings, etc.’* (P/SM).

##### Drivers and incentives for different groups

Researchers, policy makers, and practitioners were observed to have different needs, interests, and values systems that impact on scaling up processes: As a policy maker observed: *‘Policy makers were trying to find the best range of tools to improve population health…’* (PM). It appeared that practitioners/service managers were driven by similar motivations as policy makers, but had a greater focus on meeting local community and stakeholder needs in scaling up processes. A practitioner observed that researchers often have a different motivation for involvement in scaling up processes: ‘*The drivers are attracting funding and publishing papers not what the research leads to in a practical sense. I don’t know how often researchers think about how soon their research is going to be used or if in fact, it will be used’* (P/SM). This observation was confirmed by a researcher who said: *‘As a researcher, we’re not rewarded for it in any way really. So that’s another factor because everything we’re rewarded on is just the old metrics and there’s no money in any grant to do it…’* (R).

## Discussion

This is the first study to provide an analysis of how decisions to scale up population health interventions have actually occurred across a range of projects in developed country contexts. The study illustrates that decisions to scale up interventions are typically subject to iterative policy or practice-based decision-making processes, frequently involving engagement with internal and external stakeholders. Policy makers generally lead these processes, but are subject to decisions by fund holders and political leaders, who must be convinced of the relevant merits of action before any action can proceed; a theme that is consistent with the global health literature [12,22].

This study’s findings indicate that policy makers, practitioners, and researchers have different but complementary roles to play in scaling population health interventions. Consistent with observations of health policy processes in previous research [16,17,23], respondents in this study reported that policy makers played a lead role in shaping priorities, securing resources and solidifying political and broader stakeholders support for large-scale action [16,17]. Respondents also noted that policy makers generally made decisions about how to implement scaling up processes based on a variety of factors, including the available evidence. As Brownson et al. [16] observed in the United States, even in the light of sound scientific data, ideas are sometimes not ready for policy action, due to lack of public support or competing interests.

Practitioners/service managers in this study appeared to perform similar stakeholder management functions to policy makers, but on a local level. Practitioners described being at the ‘coal face’ of many scaling up processes, having a proximal role in facilitating practice change through stakeholder engagement, partnership building, and service development, and this contribution to scaling up may sometimes precede larger scale up, through testing of promising interventions on a local level prior to further expansion.

While researchers provided information and advice to decision makers based on their assessments of the available evidence or the findings of their research, their greatest contribution to decisions about whether to and how to scale up interventions, was their influence on the way decision makers thought about issues, often communicated through their ongoing links with policy makers. As Brownson et al. [16] suggest, to achieve influence, researchers need to be aware of policymakers’ concerns and windows of opportunity to influence policy and practice, through active involvement and interchange of ideas. While there are legitimate reasons for researchers to maintain some level of separation from policymaking and implementation [16], population health research will have the greatest impact when its production and application are viewed as a shared responsibility between researchers and decision makers [16,24].

Co-production of research between researchers and end users of research such as policy makers and practitioners involves a different way of working in all parts of the research process [25], particularly when scaling up population health interventions. This process ideally involves end user involvement from the inception of research, starting with shaping of research questions, jointly making decisions on methodology, involvement in data collection, and tools development, and ends with the interpretation and dissemination of findings [25]. There is some evidence that, when this occurs, research findings are more likely to be relevant to end users and more likely to be used in policy and practice [24]. Co-production of research requires effective communication and exchange between researchers, policy makers, and practitioners. Collaborative mechanisms that facilitate exchange include the establishment of policy relevant research centres [26], collaborative research grants [27], communities of practice [24], and the use of knowledge brokers [28].

An important observation by respondents in this study, again consistent with previous research [3,16,17,29], was that researchers and policy makers/practitioners have different needs, value systems, and incentives. Effective, efficient, and timely implementation of interventions in a fashion that is sensitive to key stakeholders’ interests and political recognition were primary drivers for policy makers and practitioners [16,23]. While for researchers, support for scaling up of evidence-based interventions may not be particularly important, as research funding and publications were key drivers. Core performance metrics of individual researchers and research groups are usually based on the number of publications, grants, postgraduate research students, and the amount of competitive research funding [29].

Encouragingly, interviews confirmed that evidence in many forms, including research evidence, was important to underpin many decisions related to scaling up population health interventions. Respondents’ descriptions of how evidence informed scaling up processes, that is, research being only one of many influences on decision making, appear to echo the use of research evidence in broader policy making processes previously described in the literature [17,30-33].

An interesting observation was that locally-generated research evidence was highly valued by policy makers and practitioners as it was perceived to be contextually relevant and more likely to translate into practice. Local evidence of efficacy or evidence from local pilots or replication studies was thought to be particularly persuasive. However, it is important to note that policy makers and practitioners in this study described using a ‘body of evidence’, rather than any single research study, as the empirical basis for scaling up an intervention. In most cases, decision makers noted that there were large gaps in the available evidence. They often described the need to search the grey literature and other sources of information, such as parallel evidence from other settings, and rely on practitioner knowledge and expert advice to fill in these gaps. Echoing these findings, recent interviews with 38 Australia policy makers conducted by Campbell et al. [34] found that the most common reason for not using research in policy was the absence of appropriate and/or relevant research. The study found that while information about the large scale implementation of programs is rarely published, scaling up is a frequent real world occurrence, often relying on imperfect evidence [6]. There remains a paucity of policy and practice relevant forms of evidence, particularly intervention research with detailed information on costs and implementation issues in the published literature [3,5], or in local settings. An increasing number of policymakers, research funders, and researchers argue that there is an urgent need for high-quality studies assessing mechanisms by which more widespread intervention adoption and reach can be achieved [35-37].

The costs of scaling up interventions are fundamental to decisions about public health program implementation [38,39]. Costing of an intervention identifies whether the various arms of a program are receiving money as was intended in the original plan and underpins any subsequent economic evaluation [40]. Despite its value, this information is generally absent from research reports and in particular published intervention studies [6,41]. Given the importance of economic data to informing scaling up processes [6], the field should be encouraged to collect and publish data on intervention costs and, where feasible, cost effectiveness of interventions. Encouragingly, there are a number of recent international efforts aimed at facilitating more transparent reporting of intervention effectiveness and associated costs [42], namely, the Consolidated Standards of Reporting Trials (CONSORT) statement [43] and the Transparent Reporting of Evaluations with Non-randomized Designs (TREND) Statement [44]. These statements require authors of reports and journal articles to follow checklists that indicate the type of information required for research consumers to more readily use and interpret study findings. Although they have been established to improve the reporting of research, they also provide researchers with a framework by which to design the primary research itself [42]*.*

Respondents across occupational groupings in the sample noted the tension between program fidelity, that is the extent to which the implementation of the intervention is consistent with intervention protocols previously found to be effective [45] and the counter-acting pressure of adaptation, the adjustment of an intervention for different target populations, localities, and organizational factors [46]. Implementing at scale often requires substantial simplification of the original model, since the resources necessary for intensive implementation of the intervention at scale are often not available in real world settings [8,11,12]. Attaining the right balance that fits the context and circumstances, but yet retains the effective ingredients, is vital to the success of scaling up efforts [6,46,47]. It is important to continue to monitor intervention effectiveness when interventions are simplified and/or adapted as part of scaling up processes [6,14].

Overall, this study confirms that a key challenge for the field of population health is to facilitate the timely provision of policy and practice relevant research evidence into scaling up decision processes. There is opportunity for more co-production of research that directly informs efforts to scale up population health action. This study also reinforces the importance of funding agencies and journals supporting the generation and publication of such applied research.

The study findings are based on the substantial experience of senior policy makers, practitioners, and researchers, and provide a sound empirical basis for understanding the process of scaling up population health action in real world developed country contexts. This study also builds on the previous scalability research [6] by providing additional rigorous scrutiny and in-depth commentary, which confirmed how a range of considerations related to the appropriateness of an intervention for scaling up, are actually taken into account.

In summary, to be appropriate for scaling up, population health interventions generally required evidence of effectiveness, had the potential for substantially expanded reach and system adoption, had evidence showing that they were acceptable to the target groups and settings, and could be delivered at an acceptable and sustainable cost.

### Limitations and further research

Though this is the first study to comprehensively examine how scaling up decision-making processes occur in the real world across a range of projects and settings, it did not examine the appropriateness of particular policies or programs for scaling up or otherwise. This study engaged a small number of expert participants, mainly from Australia, though it is important to note that a number of respondents were from Asia, Europe, and the United States and were recognized as international experts in their respective fields of expertise. While a larger sample or different set of respondents may have generated some differing views, the consistency, rigor, and detail of responses to the interviews, as well as the substantial experience of the experts lend confidence to the results. The considered approach taken in respondent selection, high response rate, and strong contributions from all respondents also add weight to the validity of the findings. There is, however, merit in determining if the findings identified amongst this select sample of experts can be replicated with a larger and more representative sample of policy makers, practitioners, and researchers.

## Conclusions

In order to achieve population-wide health improvements, population health interventions that have been found to be efficacious in research should be considered for widespread implementation. A better understanding of how decisions to scale up interventions are made in practice, the role of evidence, and the respective roles of policy makers, practitioners, and researchers have played in such processes can inform future intervention research design, intervention development, and scaling up decisions more broadly.

At least part of the reason for the uneven dissemination of intervention research findings into population health practice appears to be lack of information relevant to the needs of decision makers when they are managing scaling up processes. The field is encouraged to address key evidence gaps to better inform scaling up processes, particularly publishing more intervention research that provides data on the effectiveness, reach, and costs of operating at scale as well as key service delivery issues including acceptability and fit of interventions and delivery models with the local context. Addressing these evidence gaps would advance the relevance and ultimately usability of research for policymakers and practitioners charged with scaling up population health action.

## Competing interests

The authors declare that they have no competing interests.

## Authors’ contributions

AJM’s contribution to this paper included conceptualizing the study, developing data collection instruments, conducting content and thematic analysis of surveys and interviews, led the design of the manuscript, drafted and edited the manuscript taking into account input from co-authors and comments from journal peer reviewers. LK and CR contributed to the study design, design of the paper, reviewing and editing the manuscript. RN conducted interviews, contributed to reviewing and editing the manuscript. LW, AB and SR contributed to reviewing and editing the manuscript.

## References

[B1] Productivity Commission Strengthening Evidence-Based Policy in the Australian Federation, Volume 2: Background Paper 2010 Canberra (AUST): Productivity Commission

[B2] Balas EA Boren SA Yearbook of Medical Informatics: Managing Clinical Knowledge for Health Care Improvement 2000 Stuttgart, Germany 27699347

[B3] Sanson-Fisher RW Campbell EM Htun AT Bailey LJ Miller CJ We are what we do: research outputs of public health Am J Prev Med 2008 35 4 380 385 10.1016/j.amepre.2008.06.039 18687567

[B4] McKeon R Strategic Review of Health and Medical Research in Australia – Better Health Through Research 2013 Canberra Commonwealth of Australia [http://www.mckeonreview.org.au/]

[B5] Milat AJ Bauman A Redman S Curac N Public health research outputs from efficacy to dissemination: a bibliometric analysis BMC Public Health 2011 11 934 10.1186/1471-2458-11-934 22168312 PMC3297537

[B6] Milat AJ King L Bauman A Redman S The concept of scalability: increasing the scale and potential adoption of health promotion interventions into policy and practice Health Promot Int 2013 28 3 285 298 10.1093/heapro/dar097 22241853

[B7] Milat AJ Newson R King L Rissel C Wolfenden L Bauman AE Redman S Increasing The Scale and Adoption of Public Health Interventions: A Guide for developing a Scaling Up Strategy 2014 North Sydney: NSW Ministry of Health

[B8] Kohl R Cooley L Scaling Up – A Conceptual and Operational Framework 2003 Washington, DC: Management Systems International

[B9] Milat AJ King L Bauman A Redman S Scaling up health promotion interventions: an emerging concept in implementation science Health Promot J Austr 2011 22 238 22497071 10.1071/he11238

[B10] World Health Organization, ExpandNet Nine Steps for Developing a Scaling-Up Strategy 2010 Geneva: WHO

[B11] Norton W Mittman B Scaling-Up Health Promotion/Disease Prevention Programs in Community Settings: Barriers, Facilitators, and Initial Recommendations Report Submitted to the Patrick and Catherine Weldon Donaghue Medical Research Foundation 2010 [http://donaghue.org/wp-content/uploads/Final-Scaling-Up-Report.pdf]

[B12] Yamey G Scaling up global health interventions: a proposed framework for success PLoS Med 2011 8 6 e1001049 10.1371/journal.pmed.1001049 21738450 PMC3125181

[B13] Mangham LJ Hanson K Scaling up in international health: what are the key issues? Health Policy Plan 2010 25 2 85 96 10.1093/heapol/czp066 20071454

[B14] Larson CP Koehlmoos TP Sack DA Scaling Up of Zinc for Young Children (SUZY) Project Team: Scaling up zinc treatment of childhood diarrhoea in Bangladesh: theoretical and practical considerations guiding the SUZY Project Health Policy Plan 2012 27 2 102 114 10.1093/heapol/czr015 21343236

[B15] Victora CG Barros FC Assunção MC Restrepo-Méndez MC Matijasevich A Martorell R Scaling up maternal nutrition programs to improve birth outcomes: a review of implementation issues Food Nutr Bull 2012 33 2 S6 S26 22913105 10.1177/15648265120332S102

[B16] Brownson RC Royer C Ewing R McBride TD Researchers and policymakers: travelers in parallel universes Am J Prev Med 2006 30 2 164 172 10.1016/j.amepre.2005.10.004 16459216

[B17] Bowen S Zwi AB Pathways to “evidence-informed” policy and practice: a framework for action PLoS Med 2005 2 7 e166 10.1371/journal.pmed.0020166 15913387 PMC1140676

[B18] Fink A Kosecoff J How to conduct surveys: a step-by-step guide 1985 London: Sage Publications

[B19] Kokko S Kannas L Villberg J The health promoting sports club in Finland – a challenge for the settings-based approach Health Promot Int 2006 21 3 219 229 10.1093/heapro/dal013 16684782

[B20] Boyatzis RE Transforming Qualitative Information: Thematic Analysis and Code Development 1998 Thousand Oaks, CA: SAGE Publications

[B21] Braun V Clarke V Using thematic analysis in psychology Qual Res Psychol 2006 3 2 77 101 10.1191/1478088706qp063oa

[B22] Rani M Nusrat S Hawken L A qualitative study of governance of evolving response to non-communicable diseases in low-and middle-income countries: current status, risks and options BMC Public Health 2012 12 1 877 10.1186/1471-2458-12-877 23067232 PMC3487912

[B23] Bowen S Zwi A Sainsbury P What evidence informs government population health policy? Lessons from early childhood intervention policy in Australia NSW Public Health Bulletin 2005 16 180 184 16778918 10.1071/nb05050

[B24] Milat AJ Laws R King L Newson R Rychetnik L Rissel C Bauman AE Redman S Bennie J Policy and practice impacts of applied research: a case study analysis of the New South wales health promotion demonstration research grants scheme 2000–2006 Health Res Policy Syst 2013 11 1 5 10.1186/1478-4505-11-5 23374280 PMC3621590

[B25] Graham ID Tetroe J How to translate health research knowledge into effective healthcare action Healthcare Q 2007 10 3 20 22 10.12927/hcq..18919 17632905

[B26] Milat A King L Bauman A The physical activity, nutrition and obesity research group: fostering population health research in NSW NSW Public Health Bulletin 2011 22 2 13 14 10.1071/NB10059 21527073

[B27] NHMRC Partnerships for Better Health Australian Government National Health and Medical Research Council http://www.nhmrc.gov.au/grants/apply-funding/partnerships-better-health]

[B28] Armstrong R Waters E Crockett B Keleher H The nature of evidence resources and knowledge translation for health promotion practitioners Health Promot Int 2007 22 3 254 260 10.1093/heapro/dam017 17596543

[B29] Taylor J The impact of performance indicators on the work of university academics: evidence from Australian universities High Educ Q 2001 55 1 42 61 10.1111/1468-2273.00173

[B30] Kothari A Rudman D Dobbins M Rouse M Sibbald S Edwards N The use of tacit and explicit knowledge in public health: a qualitative study Implement Sci 2012 7 1 20 10.1186/1748-5908-7-20 22433980 PMC3325865

[B31] Dobbins M Rosenbaum P Plews N Law M Fysh A Information transfer: what do decision makers want and need from researchers Implement Sci 2007 2 20 12 17608940 10.1186/1748-5908-2-20 PMC1929120

[B32] Jacobs JA Dodson EAE Baker EAE Deshpande AD Brownson RC Barriers to evidence-based decision making in public health: a national survey of chronic disease practitioners Public Health Rep 2010 125 5 736 20873290 10.1177/003335491012500516 PMC2925010

[B33] Kothari A Birch S Charles C “Interaction” and research utilisation in health policies and programs: does it work? Health Policy 2005 71 1 117 125 10.1016/j.healthpol.2004.03.010 15563998

[B34] Campbell DM Increasing the use of evidence in health policy: practice and views of policy makers and researchers Aust New Zealand Health Policy 2009 6 21 10.1186/1743-8462-6-21 19698186 PMC2739528

[B35] NIH Dissemination and Implementation Research in Mental Health PA-02-131 [http://www.nimh.nih.gov/about/organization/dsir/services-research-and-epidemiology-branch/dissemination-and-implementation-research-program.shtml]

[B36] Rubenstein LV Pugh J Strategies for promoting organizational and practice change by advancing implementation research J Gen Intern Med 2006 21 S58 S64 16637962 10.1111/j.1525-1497.2006.00364.x PMC2557137

[B37] Catford J Advancing the ‘science of delivery’ of health promotion: not just the ‘science of discovery’ Health Promot Int 2009 24 1 5 19171666 10.1093/heapro/dap003

[B38] Johns B Baltussen R Accounting for the cost of scaling up health interventions Health Econ 2004 13 11 1117 1124 10.1002/hec.880 15386683

[B39] Bishai D McQuestion M Chaudhry R Wigton A The costs of scaling up vaccination in the world’s poorest countries Health Aff 2006 25 2 348 356 10.1377/hlthaff.25.2.348 16522576

[B40] Norman R Haas M Issues in the Costing of Large Projects in Health and Healthcare 2009 North Sydney: NSW Department of Health

[B41] Neville L O’Hara B Milat AJ Computer-tailored nutrition interventions targeting adults: a systematic review Health Educ Res 2009 24 4 699 720 10.1093/her/cyp006 19286893 PMC2706490

[B42] Armstrong R Waters E Moore L Riggs E Cuervo LG Lumbiganon P Hawe P Improving the reporting of public health intervention research: advancing TREND and CONSORT J Pub Health 2008 30 1 103 109 10.1093/pubmed/fdm082 18204086

[B43] Montgomery P Grant S Hopewell S Macdonald G Moher D Michie S Mayo-Wilson E Protocol for CONSORT-SPI: an extension for social and psychological interventions Implement Sci 2013 8 1 99 10.1186/1748-5908-8-99 24004579 PMC3766255

[B44] Des Jarlais DC Lyles C Crepaz N TREND Group Improving the reporting quality of nonrandomized evaluations of behavioral and public health interventions: the TREND statement Am J Public Health 2004 94 3 361 366 10.2105/AJPH.94.3.361 14998794 PMC1448256

[B45] Mowbray CT Holter MC Teague GB Bybee D Fidelity criteria: development, measurement, and validation Amer J Evaluation 2003 24 3 315 340 10.1177/109821400302400303

[B46] Shen J Yang H Cao H Warfield C The fidelity–adaptation relationship in non-evidence-based programs and its implication for program evaluation Evaluation 2008 14 4 467 481 10.1177/1356389008095488

[B47] Backer TE Finding the Balance: Program Fidelity and Adaptation in Substance Abuse Prevention: A State-of-the-art Review 2001 Rockville: US Department of Health and Human Services Center for Substance Abuse Prevention

